# Comparison of postoperative visual performance between trifocal intraocular lens and monofocal intraocular lens

**DOI:** 10.15537/smj.2023.44.5.20220833

**Published:** 2023-05

**Authors:** Wanping Zhang, Ting Peng, Xi Cheng, Chen Wang, Xiangyun Lv, Siting Liang, Jun Hu

**Affiliations:** *From the Department of Ophthalmology (Zhang, Cheng, Hu), Tongji Hospital, Huazhong University of Science and Technology; and from the Department of Cataract (Zhang, Peng, Lv); from the Department of Imaging (Liang), Aier Eye Hospital of Wuhan University; and from the Department of Ophthalmology (Wang), Union Hospital, Huazhong University of Science and Technology, Wuhan, China.*

**Keywords:** contrast sensitivity, defocus curve, modulation transfer function, Strehl ratio, dysfunctional lens index

## Abstract

**Objectives:**

To compare the subjective and objective visual quality more comprehensively after surgery of the commonly used multifocal intraocular lenses (IOL) and monolocal IOL implants through long-term systematic clinical observation, providing reference and basis for clinical application.

**Methods:**

Non-randomized controlled trial. A total of 91 (138 eyes) patients between June 2020 and December 2020 were implanted trifocal IOL or monofocal IOL after phacoemulsification in a tertiary class hospital in Wuhan. Monocular testing 3 months after surgery included best-spectacles corrected and uncorrected visual at distant, intermediate, and near vision; spherical equivalent (SE); defocus curve; modulation transfer function (MTF); dysfunctional lens index (DLI); Strehl ratio (SR); mesopic contrast sensitivity function; quality-of-life, spectacles independence, visual disturbance, and surgical satisfaction surveys 3 months post-surgery.

**Results:**

There was statistically better uncorrected vision acuity with trifocal IOLs in all range, while monofocal IOL had statistically better mesopic contrast sensitivity at specific spatial frequencies and statistically worse defocus curves, spectacles independence, and surgical satisfaction. The trifocal IOL performed better in subjective quality of vision and life and spectacles independence questionnaires, and the objective quality of vision had no statistical significance.

**Conclusion:**

Compared to monofocal IOL, trifocal IOL could provide a full range of clear vision for the majority of patients with simple cataracts, improve the rate of spectacles independence and patient satisfaction. And the objective quality of vision did not show any difference.

**C**ataract is the first reversible blindness-causing eye disease worldwide; however, the underlying pathogenesis is yet inconclusive. Albeit age and some genetic factors^1,2^ are the main causes of cataract formation. With the overall improvement in the social, economic, and medical development, the patients have put forward high requirements for the postoperative effect of cataract. Spectacles independence and younger visual experience have become the pursuit of the elderly. Decades ago, the primary goal of cataract surgery was sight rehabilitation. However, with the development of ophthalmic surgical equipment and intraocular lens (IOL) material science, especially the emergence and popularization of various functional IOLs, such as various multifocal intraocular lens (MIOL), diffractive apodized bifocal toric IOL, diffractive extended depth of focus IOL (ReSTOR,^3^ Tecnis ZMA00,^4^ ReZoom,^4^ Lentis Mplus MF30,^5^ and Tecnis ZM900,^5^ refractive cataract surgery has been able to provide clear, comfortable, and high-quality vision based on sight rehabilitation for patients.^6,7^

Although patients can have good distance vision after the implantation of monofocal IOL, their vision in intermediate and near remain poor due to the loss of accommodation. Most of the individuals are spectacles independent, but the overall contentment is not high. The new IOL can improve the postoperative distance, intermediate, and near vision while providing depth of focus and patient satisfaction. Previous studies^8^ have shown that multifocal intraocular lens provided good optical quality and spectacle independence. Furthermore, in a questionnaire sent publicly to ophthalmologists, 67.7% of them answered that they were willing to implant new IOL.^9^ Multifocal intraocular lens has been widely used and studied worldwide. After implantation, it provides the patients with excellent distance, intermediate, and near vision;^8,10^ however, some visual interference, such as xerophthalmia, ametropia, glare, and halation, from high spectacles independence cannot be ignored.^11^ Some studies^12-16^ have shown that MIOL implantation would bring low contrast sensitivity and visual interference, while some researchers speculated that the contrast sensitivity of MIOL implantation is similar to monofocal IOL^17^ or it may decrease in the early stage and have the same contrast sensitivity as monofocal IOL after 3 months.^18^

Currently, many late-model inspection instruments are putting into clinical use. This study uses multiple inspection instruments to compare the subjective and objective visual quality more comprehensively after surgery of the commonly used multifocal IOL and monolocal IOL implants through long-term systematic clinical observation, providing reference and basis for clinical application.

## Methods

### Search method used to find prior related research

Conduct relevant literature searches on websites such as PubMed, Web of Science, SCI-HUB, with the search keywords including MIOL, contrast sensitivity (CS), and modulation transfer function (MTF); Dysfunctional lens index (DLI), and so on.

### Patient enrollment

After approval by the ethics committee, 91 (138 eyes) patients between June 2020 and December 2020, aged >40-years-old, with age-related cataracts, were enrolled consecutively in a non-randomized controlled trial comparing AT LISA tri 839MP (Carl Zeiss Meditec, Jena, Germany) (A group; n=65) and Mi60 (Bausch and Lomb, New York, American) (B group; n=75) implantation in a tertiary class hospital in Wuhan (Table 1). No statistical difference was observed in age, axial length, endothelium, and vision between groups A and B. The inclusion criteria were as follows: the need for regular follow-up, the difference between the simulated keratometry reading (Simk) and total corneal refractive power (TCRP) <0.5 diopter (D), and the axial difference <10°; the ratio of back to front corneal radii (B/F ratio) is around 82%; the total corneal irregular astigmatism in 4 mm area <0.3 µm; <-6.0 D of spherical; or <26 mm of axial length; scotopia pupil size <6 mm and photopic pupil size >2.5 mm; <0.2 µm of the spherical aberration of monofocal IOL, >0.2 and <0.4 µm of trifocal IOL; <0.5 µm of angle kappa and <0.5 µm of angle alpha. The exclusion criteria included systemic diseases that affect eyesight, history of eye operation, history of eye trauma, >1.25 D of corneal astigmatism with-the-rule, >0.75 D corneal astigmatism against-the-rule, glaucoma, congenital cataract. The demographics of the subjects are detailed in Table 1.

**Table 1:** - Characteristics of study participants.^+^

Characteristics	Group A	Group B	*P*-value
Number	42	49	
Gender (female/male)	19/23	25/24	
Eyes (female/male)	34/31	38/35	
Age (years)*	57.14±14.67	60.42±12.27	0.395
Endothelial cells (pcs/mm^2^)	2623.07±302.75	2675.05±258.05	0.677
Axis (mm)	23.94±1.11	23.84±1.13	0.867
UCVA	0.23±0.25	0.32±0.24	0.694
BCVA	0.40±0.31	0.39±0.27	0.616

^*^ Person’s age at the time of eye surgery,

+All comparisons were not significant (*p*>0.05),

UCVA: uncorrected vision acuity,

BCVA: best-corrected vision acuity

### Surgical procedure

All the operations were performed by the same doctor. The patients underwent small incision phacoemulsification with an incision at the steep meridian and a central continuous curvilinear capsulorhexis, <5.5 mm in diameter.

### Intraocular lens selection, IOL power calculations, and targeted refraction

A detailed medical history inquiry and explanation of postoperative results were essential before MIOL implantation.^19^ The optical system of trifocal IOL is different from that of a natural lens. Only when patients understood the difference and were willing to adapt for the long term, trifocal IOL implantation could be considered.

Barrett universal II formula was used for all patients. Emmetropia was the targeted refraction for AT LISA tri 839MP, and -0.25D could be used if patients had special requirements for near vision. For Akreos Mi60, according to the patients’ needs, refraction was targeted between -0.25 D and -0.75 D, while monofocal IOL did not have good near vision and required close work.

### Subjective visual quality

Uncorrected vision acuity (UCVA) at a distance (5 m), intermediate (80 cm), and near (40 cm), spherical equivalent (SE), and best-corrected vision acuity (BCVA) at a distance and near were recorded. Also, the best-corrected visual at intermediate was not recorded. We averaged the logarithm of the minimum angle of resolution (logMAR) visual acuities. The equivalent spherical lens was recorded 3 months after the operation. The postoperative testing was on day 7 and months 1 and 3. In the best-corrected vision, contrast sensitivity (CS) was measured at 1.5, 3, 6, 12, and 18 cycles per degree (cpd) using CSV-1000 (Vector vision Inc., Greenville, USA). The defocus curves under monocular UCVA and area-of-focus metric were measured.^20^ The postoperative testing windows were months 1 and 3. Visual function index-14 questionnaires have been validated previously with respect to spectacles independence, visual disturbance, and surgical satisfaction surveys at 3 months after the implantation.

### Objective visual quality

Modulation transfer function (MTF) average height, dysfunctional lens index (DLI), and Strehl ratio (SR) were recorded at 3 months after implantation using the iTrace aberrometer (Tracey Technologies Co., Houston, USA). MTF values were measured at spatial frequencies of 5, 10, 15, 20, 25, and 30 cpd at 3-mm pupil diameter.

### Statistical analysis

The SPSS Statistics for Windows, version 26.0 (IBM Corp., Armonk, N.Y., USA) was used for statistical analyses. Confirm all data distribution were normal distribution. For each parameter, the mean values and standard deviations were calculated. Independent sample t-test was used between groups A and B, and statistical significance was indicated by *p*-value of 0.05.

We followed the tenets of the Declaration of Helsinki, and obtained written informed consent from all participants prior to their inclusion in the study, and all experiments were approved by the Ethics Committee of Wuhan Aier Hongshan Eye Hospital (acceptance number: HS2020IRBKY01).

## Results

In group A, 2 patients had fundus lesions that were not detected before the operation, and 3 patients in group B were lost to follow-up after the operation. None of these 5 patients were included in the follow-up data analysis.

### Monocular vision

**Figure 1:**
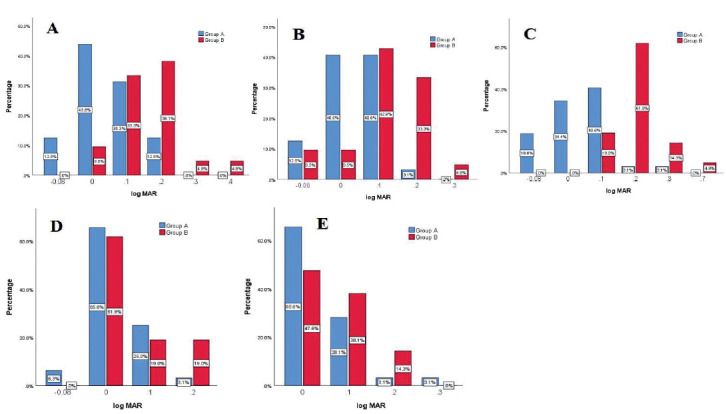
- Visual acuity distribution of each group. **A**) distance UCVA distribution of each group; **B**) intermedia UCVA distribution of each group; **C**) near UCVA distribution of each group; **D**) distance BCVA distribution of each group; **E**) near BCVA distribution of each group. UCVA: uncorrected visual acuity, BCVA: best-corrected visual acuity

Uncorrected vision acuity at distant *p*<0.001, intermediate *p*=0.006, and near vision *p*<0.001 and SE *p*<0.001 were statistically superior for group A (63 eyes), compared to group B (70 eyes) at 3 months after the surgery (Table 2). The monocular mean BCVA did not show a statistically significant difference at distant and near vision in groups A and B (*p*=0.109, 0.228) (Table 2 & Figure 1).

**Table 2:** - Distance, intermediate, and near vision at 3 months after surgery with LISA tri 839MP and Mi60.^+^

Intraocular lens	n*	Distance UCVA	Distance BCVA	Intermediate UCVA	Near UCVA	Near BCVA	SE
AT LISA tri 839MP	63	0.04±0.08	0.04±0.08	0.04±0.09	0.03±0.06	0.04±0.07	-0.46±0.30
Mi60	70	0.12±0.10	0.12±0.10	0.20±0.06	0.06±0.0	0.07±0.07	-0.92±0.40
*P*-value		<0.001	0.006	<0.001	0.109	0.228	<0.001

^*^ n represented the number of eyes followed up after surgery (one case in group A was lost to follow-up, and there were 2 cases of fundus disease in group B not found before the operation. These 3 patients were not included in the postoperative statistical analysis).

^+^ All visual acuity was expressed in logMAR,

UCVA: uncorrected visual acuity,

BCVA: best-corrected visual acuity,

SE: spherical equivalent

### Contrast sensitivity function

**Figure 2:**
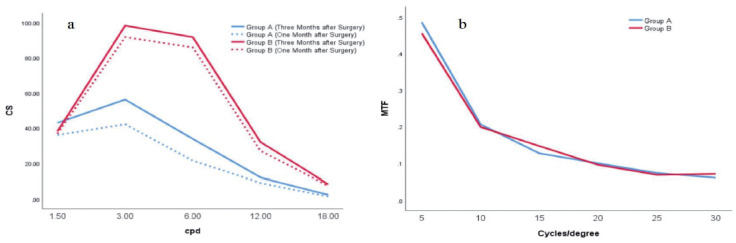
- Contrast sensitivity function and modulation transfer function (MTF) of each group after surgery. CS: contrast sensitivity, cpd: cycles per degree

Across all spatial frequencies, group B scored higher on the monocular, best spectacles corrected contrast sensitivity test except 1.5 cpd without glare than group A at 1 and 3 months (Figure 2-a), and group B had statistically better contrast sensitivity function (CSF) at 3 cpd (*p*<0.001), 6 cpd (*p*<0.001), 12 cpd (*p*<0.001), and 18 cpd (*p*=0.018) without glare. The mean values at medium and higher spatial frequencies remained elevated until 3 months postoperatively, especially in group A.

### Defocus curves and area-of-focus metric

**Figure 3:**
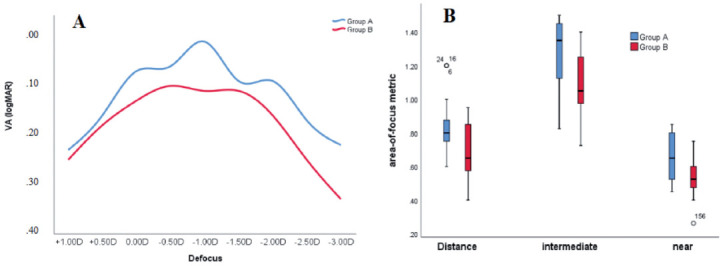
- The defocus curves and the area-of-focus metric of the 2 groups. **A**) The defocus curves of the 2 groups; **B**) the area-of-focus metric of the 2 groups. The near area was between -4.0 D and -2.0 D, corresponding to the range from 25 cm to 50 cm; the intermediate area was between -2.0 D and -0.50 D, corresponding to the range from 50 cm to 2 m; the distance area was defined as -0.50 D to +0.50 D, corresponding to the range beyond 2 m. D: diopter, VA: vision acuity, logMAR: minimum angle of resolution

Defocus curves under monocular uncorrected visual acuity showed that group A achieved higher VAs over +1.00 D to -3.00 D than group B (Figure 3). The area-of-focus analysis method revealed a statistically significant difference between groups A and B for distance area (*p*=0.002), intermediate area (*p*=0.033), and near area (*p*=0.021).

### Subjective visual quality

The VF-14-questionnaire aggregate score of group A was statistically higher than that of group B (*p*<0.001). At distance vision, 87.1% of group A patients were completely satisfied with their surgery, while those were 40.9% for group B. No patients in group A were dissatisfied, as opposed to 3 patients in group B, and one patient in group B was very dissatisfied. The complete satisfaction rates of group A were 93.5% and 93.5% at intermediate and near distance, while those for group B were 36.4% and 18.2%. Over 90% of patients in group A could have spectacles independence in all distances. For group B, the percentage of spectacles independence was 40.9% for distance, 31.8% for intermediate, and 18.2% for near vision.

Most patients did not have an obvious visual disturbance after surgery. Two patients in group A developed abnormal color vision and mild photophobia after surgery, and the symptoms were relieved 3 months post-surgery, while another patient with foreign body sensation did not show a noticeable improvement. In group B, 3 patients experienced ocular foreign body sensation and mild photophobia after surgery, which was significantly improved 3 months after the surgery; no statistically significant difference was observed between the 2 groups.

**Table 3:** - The MTF average height, SR, and DLI at 3 months after surgery with LISA tri 839MP and Mi60.

Intraocular lens	n*	MTF aver	SR and	DLI and
AT LISA tri 839MP	63	0.277±0.012	0.094±0.010	8.725±0.367
Mi60	70	0.252±0.015	0.084±0.116	8.909±0.330
*P*-value		0.207	0.500	0.717

^*^ n represented the number of eyes followed up after surgery,

MTF: modulation transfer function,

SR: Strehl ratio,

DLI: dysfunctional lens index

### Objective visual quality

The comparison of MTF average height values at the same spatial frequencies *p*=0.207, SR *p*=0.500, and DLI *p*=0.717 showed no statistically significant difference between 2 groups at 3 months post-operation (Table 2). The MTF values at 5 (*p*=0.513), 10 (*p*=0.773), 15 (*p*=0.216), 20 (*p*=0.707), 25 (*p*=0.595), and 30 (*p*=0.193) cpd spatial frequencies were different between the 2 groups, but the comparison of MTF values at the same spatial frequencies showed no statistical significance.

## Discussion

Cataract extraction combined with IOL implantation is the only effective method for the treatment of cataracts at present. Patients put forward higher requirements for the postoperative effect of cataracts to meet the needs and improve the quality of life. A previous study^21^ indicated that MIOL was a cost-effective option than monofocal IOL for patients who wish to have spectacles independence at all distances from a social and healthcare perspective. The application of MIOL made cataract surgery applicable to personalized functional IOL^22^ and beginning a new era of refractive cataract surgery.

Typically, doctors implanted monofocal IOL for patients during cataract surgery, which could provide good distance only. Thus, the patients required spectacles correction for intermediate and near vision postoperatively. The emergence of trifocal IOL makes it possible for patients to have spectacles independence after surgery. Trifocal IOL can converge parallel light into far, intermediate, and near focus and provide a good functional vision at all distances with high levels of spectacles independence and patient satisfaction. In the current study, group A (with 839MP) had better monocular UCVA in the distance, intermediate, and near vision, while BCVA had no difference.

When different types of IOLs were implanted, statistically significant differences were detected in the area-of-focus metric between groups A and B in the distance area, intermediate area, and near area. The range from 0-4.34 mm diameter center is +1.66 D, +3.33 D, with additional medium and near vision trifocal design, and that for 4.34-6 mm is +3.33 D bifocal designed with additional near vision. This design results in wider and better vision after trifocal IOL implantation, which in turn, leads to better visual function, higher satisfaction, spectacles independence, and quality of life, especially in the intermediate and near distance. The study by Tarib et al^23^ also showed that 92%, 92%, and 75% of patients with multifocal IOL implantation achieve complete spectacles independence at long, intermediate, and short distances, which was similar to the results of the current study.

Although the contrast sensitivity values of patients with scotopic and photopic vision after trifocal IOL implantation were within the normal range, Group B had significantly higher CSF under UCVA at 3 months after the operation in our study.^6^ This difference could may be caused by the design of the trifocal IOL that divided the visual acuity of macular into 3 parts: the distant, the intermediate, and the near. In the process of human eye aging, the visual cells in the macula are constantly decreasing. Coupled with the diffraction design of the trifocal IOL, the vision of the macula is dispersed. Therefore, it may not reach the threshold potential at a spatial frequency. However, the monofocal IOL has only one focus and does not distract the vision of the macula. Therefore, the contrast sensitivity of the patient after the monofocal IOL implantation was still within the normal range, necessitating additional research. Alternatively, this reduction may be related to the tilt of the trifocal IOL.^24^

Due to the diffraction design of this trifocal IOL, patients had more visual interference after implantation, especially glare and halos. However, no statistical difference was detected in the visual interference between the 2 groups. This may be related to the special design of the trifocal IOL, making it to have high light energy utilization. Interestingly, we found that 2 patients in group A had an abnormal color vision after implantation, and their vision was slightly bluish, while no patient reaction was noted in group B. This phenomenon could be attributed to the diffraction ring design of this trifocal IOL. The height of the diffraction step of IOL with different diopters determined the passage of specific diffracted light waves, while the wavelengths of light of different colors varied, resulting in abnormal color vision in some patients due to the diffraction of light waves after implantation. However, this part of the abnormality could be gradually alleviated after brain fusion and adaptation and did not affect the patient’s daily life. Nonetheless, this finding needs to be substantiated further. However, this study was limited by the sample size and the recruitment of patients from only one hospital.

In conclusion, compared to monofocal intraocular lens, the trifocal intraocular lens provides a full range of clear vision for the majority of patients with simple cataracts and improves the rate of spectacle independence and patients’ satisfaction, but reduces the patients’ contrast sensitivity and cause visual impairment. However, the objective quality of vision did not show any difference and would not significantly affect the daily life of the patients. Despite visual interference, trifocal intraocular lenses are preferred by patients. By strictly controlling the indications of trifocal intraocular lenses implantation, such as spherical aberration, the total corneal irregular astigmatism in 4-mm area, and the kappa angle, this visual interference can be reduced to a minimum and would not affect the daily life of patients.
